# Supplementary material for: Child volunteers in a women's paramilitary organization in World War II have accelerated reproductive schedules
Source: Nat Commun. 2020 May 12;11:2377. doi: 10.1038/s41467-020-15703-0 (PMC7217904; doi:10.1038/s41467-020-15703-0)
Supplement: Supplementary file 1 — Supplementary Information [file 41467_2020_15703_MOESM1_ESM.pdf]

Child volunteers in a women's paramilitary organization in World War II have accelerated reproductive schedules

Lynch et. al.

1. SUPPLEMENTARY INFORMATION

Lotta Svärd and the war

The volunteer based Lotta Svärd organization was tasked with supporting troops as nurses, air raid spotters, mess personnel and in other auxiliary capacities. By the end of the War, volunteers numbered over 282,000 women in the adult and youth corps. A large percentage of Lottas served behind the front lines in support duties, exposing themselves to a variety of risks. The ideology of the organization was developed from the core values of Home, Faith and the Fatherland.

Lotta Svärd founded the 'Lotta Girls' corps in 1931 as a tool to help increase interest in the defense of the country and to educate and train girls for later service. Joining the Lotta Girls division required girls to be at least 8 years of age and written permission from a parent or guardian. By 1939 there were 24,000 Lotta Girls which had increased to nearly 50,000 by 1943[1]. Toward the end of the war Lotta Girls aged 14-16 were often entrusted with activities which were usually reserved for adult Lottas, however, the lack of personnel led to some girls assisting nurses in military hospitals, and participating as air raid spotters and messengers. Their basic duties during the war included knitting gloves and socks for soldiers, writing letters to soldiers at the front and attending funerals of fallen soldiers.

2. SUPPLEMENTARY RESULTS

Selection bias

A persistent problem for quasi-experimental studies like this one, where members of Lotta Svärd are volunteers, is that of selection bias. In this study this can result in unmeasured differences between women who volunteered and those who did not, which may differentially affect their reproductive timing. In these models, however, we have sought to control for as much of this as possible. First, factors that previous research or theoretical considerations indicated have important impacts on reproductive outcomes, such as education or farming, were included in the models. Second, by analyzing a subset of women who had at least one full sister (see Methods: *Statistical Analysis*), we were able to take into account many family effects that can affect fertility outcomes such as family size or father absence. However, because there are still any number of potential differences that we were unable to fully take into account, we have included descriptive statistics (means and standard errors), comparing Lottas with Non-Lottas on a variety of traits (e.g. percentage with an education and number of siblings) that previous research has suggested might affect reproduction (see Supplementary materials: Table 6). These comparisons reveal some of the key advantages and disadvantages of only analyzing sisters. For example, although sisters are much closer than non-sisters on most of the analyzed traits, there is more uncertainty around the estimates.

However, we were also able to take advantage of another opportunity that these data present. Because the war separated the same population of women, we considered the different experiences of the volunteers and the non-volunteers during the war as a treatment and regarded their reproductive schedules before and after the war as pre and post-treatment conditions. Therefore, we used models of the reproductive schedules of the same women before the war began as a baseline (i.e. pre-treatment group) to which their reproductive schedules after the war (i.e. post-treatment group) can be compared. Results of models using the full sample of women (see Supplementary Materials: Table 1) show that, although young volunteers did reproduce at a younger age and also had more children before the war, these effects were considerably less pronounced than they were after the war (see 'Lotta X age' interaction in the top and bottom panels of Supplementary Materials: Table 1). The interaction between age and volunteering for length of inter-birth intervals before the war, however, was in the opposite direction, such that younger volunteers had longer inter-birth intervals (Supplementary Table 1 - middle panel, left side). After the war they were shorter (Supplementary Table 1 - middle panel, right side). For the sisters only analysis (Supplementary Materials: Table 2), these differences were even more notable. Before the war, for instance, there were no detectable differences (95% HDI overlaps with zero) in the age-based reproductive timing of Lottas and non-Lottas (see 'Lotta X Age' interaction in Supplementary Materials: Table 2 - top and middle panels, left side) and younger volunteers had fewer children overall (bottom panel). In contrast, after the war younger volunteers waited less time to reproduce (top panel, right side) and had more children overall (bottom panel, right side). Even though there was still no detectable effect of an age X volunteer interaction on inter-birth intervals (middle panel, right side), the effect is in the predicted direction. Overall the models analyzing the age-based reproductive outcomes of volunteers before the war provide additional support for **Predictions 1-3** that experiences during the war differentially affect the reproductive schedules of young volunteers.

Sensitivity analysis

It is important to note, however, that time to first reproduction and average birth intervals after the war result from a process that is different from the one that generates total postwar reproduction. Because women who never reproduced essentially have an infinite time to first reproduction and undefined mean birth intervals, non-reproductive women were excluded from these models (N=31,613 and N=2,272 respectively) (see Methods: Statistical analysis), and therefore were not shown in the results displayed in Figures 1a-b and 3a-b in the main text or in Supplementary materials Figures 1a, 2a and Supplementary Tables 1 and 2 (top and middle panels, right side). Nevertheless, it is worth considering the possible impact of excluding non-reproductive women from these analyses on the results. This is particularly important in light of the fact that women who volunteered for Lotta Svärd are more likely to remain childless (24.4%) than those who did not (20.9%) (β = 0.19 +/- 0.03, $p<0.001$)(see Supplementary Materials: Figure 5 and Tables 3 and 4). The higher likelihood of

volunteers never reproducing is primarily limited to older Lottas which increases yearly by ∼3% more for Lottas than non-Lottas (Age X Lotta interaction: β = 0.03 +/- 0.03, p<0.001)(see Supplementary materials: Figure 5). This higher probability of Lottas failing to reproduce remains significant (β= 0.15 +/-0.03 , p<0.001) after correcting for age (β= 0.10 +/- 0.001 , p<0.001) but this is not particularly surprising because mothers with dependent children at home are less likely to volunteer[2]. Nevertheless, because the likelihood of never reproducing and age were both seen to affect the hypothesized relationship among age, volunteering, and reproductive rate, we conducted sensitivity analysis to determine how excluding non-reproductive women from the first two models — time to first reproduction and average birth intervals — may have affected our results[3]. For these analyses, we ran a Cox survival model and included all non-reproductive women as right-censored observations at 25+ (the interviews were conducted in 1970 which was 25 years after the end of the war and no further data was collected after this point), rather than excluding them. These survival models produced results that were similar to our main Bayesian models and did not have substantial effects on these results (see Supplementary Materials: Table 3).

Effect of exposure to male soldiers

To determine whether these effects are driven by greater exposure to mortality or by greater exposure to men (i.e. soldiers), we also analyzed the effect of being married vs. being single before the war began on the reproductive schedules for a subset of Lotta girls whose year of marriage was known (see Methods). The results of models which include the dummy variable 'married before the war' show that the main effect - that young Lottas have accelerated reproductive schedules - still holds even after entering both 'married before the war' and the interaction between being 'married before the war' and being a 'volunteer'. However, it is important to note that the interaction between being married before the war and being a volunteer was also a significant and *positive* predictor of faster reproductive schedules (see Supplementary Materials: Table 7). Although this does suggest that greater exposure to men could play a role in these results, this effect could also indicate that the effect of war is simply stronger on single women.

Lotta type

Another small subset of the women who identified themselves as being members of Lotta Svärd also reported their division (N=2,580). We categorized these divisions into two groups that we presumed to be more and less exposed to combat (see Methods). Summary statistics show that young Lottas (i.e. under age 25 in 1945) who were differentially exposed to combat have similar reproductive rates and total reproductive output while older Lottas (i.e. over age 25 in 1945) who were *more* exposed to combat tend to have slower reproductive schedules (see Supplementary materials Table 5). Models were also run using all of the same covariates we used in the main models (see Supplementary Materials Table 1) but limiting the sample to only volunteers whose role in Lotta Svärd was known (N=2,580) and a dummy variable indicating exposure to combat [1=exposed, 0=less exposed]. Although these results revealed a trend in the hypothesized direction (i.e. younger volunteers in areas that were presumed to have more exposure to combat had somewhat faster reproductive schedules), the interaction term between age and exposure to combat was not significant for any of the dependent variables measuring post-war reproductive rate.

Supplementary References:

1. Nevala, Seija-Leena. (2007) Lottatytöt ja sotilaspojat. 185 pp. Minerva, Helsinki.
2. Lynch R, Lummaa V, Panchanathan K, Middleton K, Rotkirch A, Danielsbacka M, O'Brien D, Loehr J. (2019) Integration involves a trade-off between fertility and status for World War II evacuees. Nature human behaviour. 2019 Apr;3(4):337.
3. McElreath, R. (2018). Statistical rethinking: A Bayesian course with examples in R and Stan. Chapman and Hall/CRC.

FIGURES AND TABLES

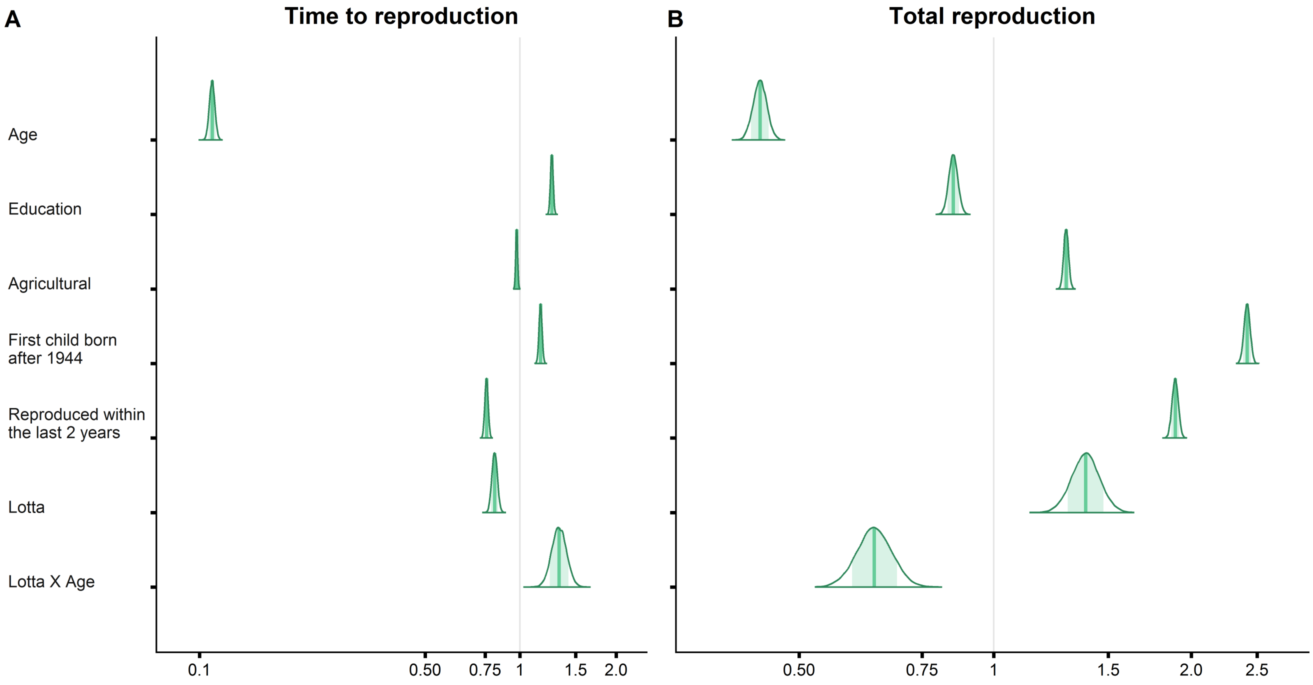

Supplementary Figure 1a-b: Posterior distributions for models run on full sample. Younger Lottas wait less time to reproduce (A) and have more children (B) after the war ends in 1945. Distributions display the entire distribution of 6000 sample draws from the posterior distribution for each parameter entered into the model. Parameter estimates have been converted to odds ratios (X-axis) (i.e. the proportional change in the outcome induced by each predictor). See Table 1 (right panels, top and bottom) for exact parameter estimates, 95% Highest Density Intervals and odds ratios for these predictors.

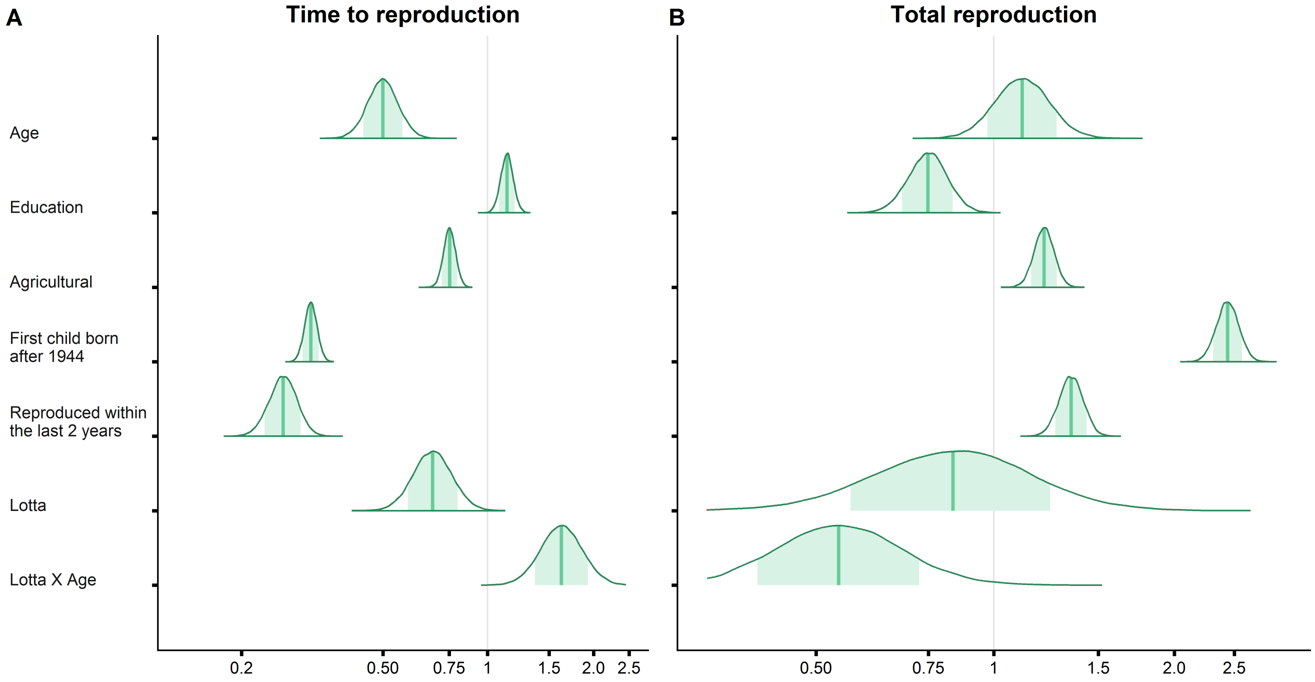

Supplementary Figure 2a-b: Posterior distributions for models run on sisters only. Younger Lottas wait less time to reproduce (A) and have more children (B) after the war ends in 1945. Distributions display the entire distribution of 6000 sample draws from the posterior distribution for each parameter entered into the model. Parameter estimates have been converted to odds ratios (X-axis) (i.e. the proportional change in the outcome induced by each predictor). See Table 2 (right panels, top and bottom - after the war) for exact parameter estimates, 95% Highest Density Intervals and odds ratios for these predictors.

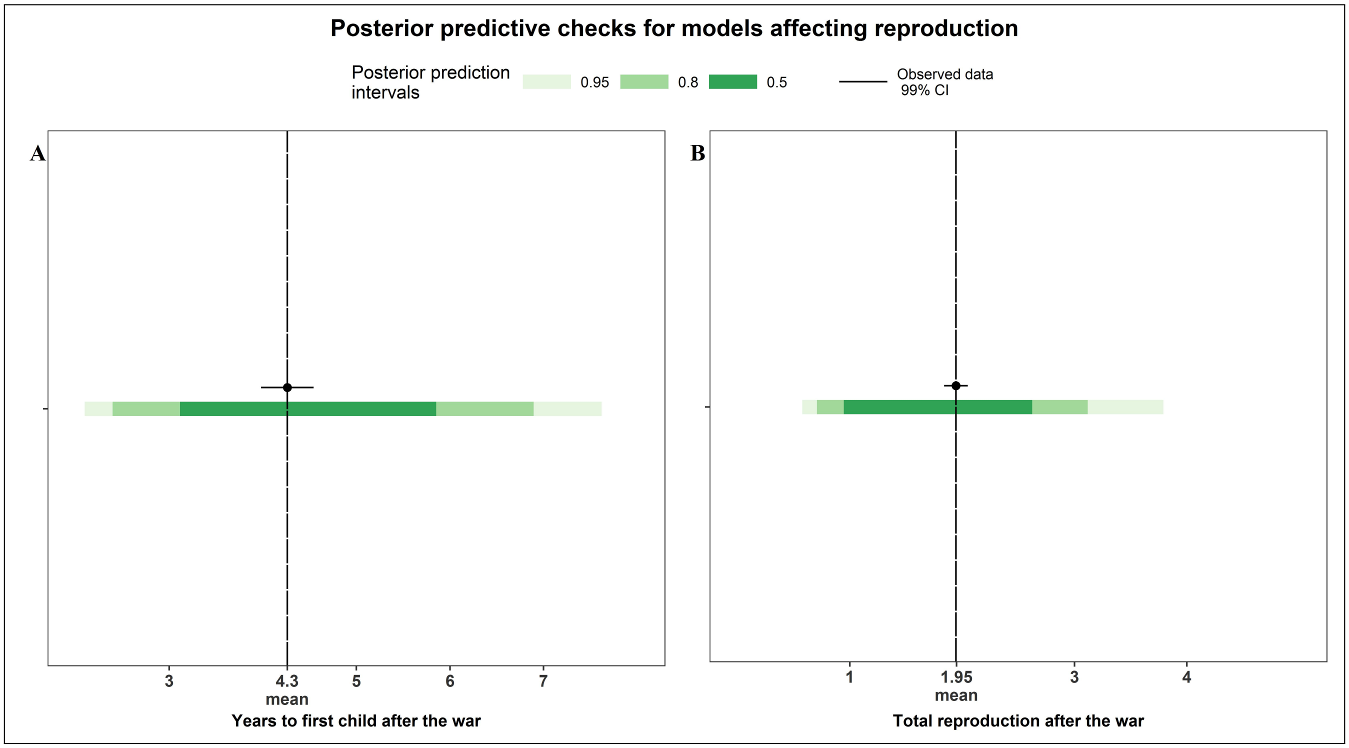

Supplementary Figure 3a-b: Posterior predictive check for models models run on full sample (A) time to reproduction and (B) total postwar reproduction: Model prediction credibility intervals (95%, 80% and 50%) in shades of green compared to the mean (black circle) and +/- 3 standard deviations (black segment) of the observed data. all three models do a reasonable job of recreating the data which suggests that the models are specified correctly.

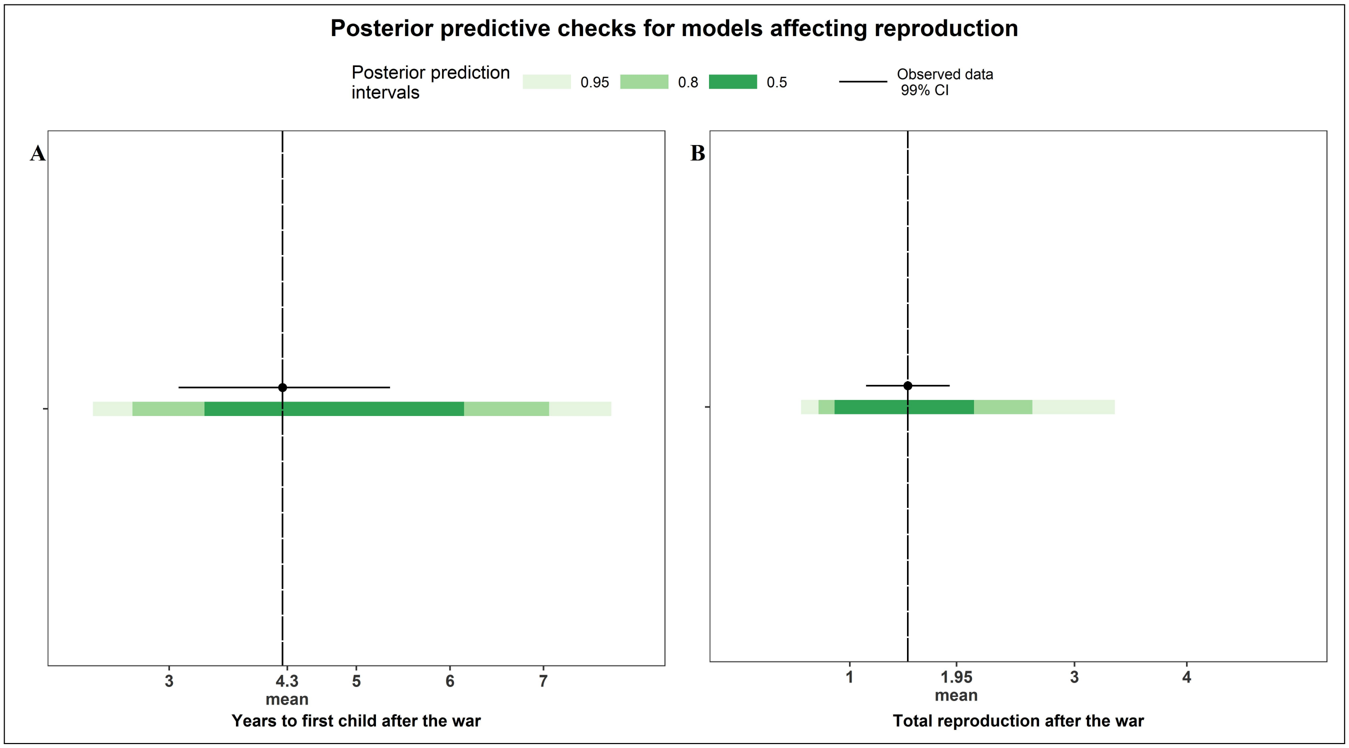

Supplementary Figure 4a-b: Posterior predictive check for models models run on sisters only (A) time to reproduction and (B) total postwar reproduction: Model prediction credibility intervals (95%, 80% and 50%) in shades of green compared to the mean (black circle) and +/- 3 standard deviations (black segment) of the observed data. all three models do a reasonable job of recreating the data which suggests that the models are specified correctly.

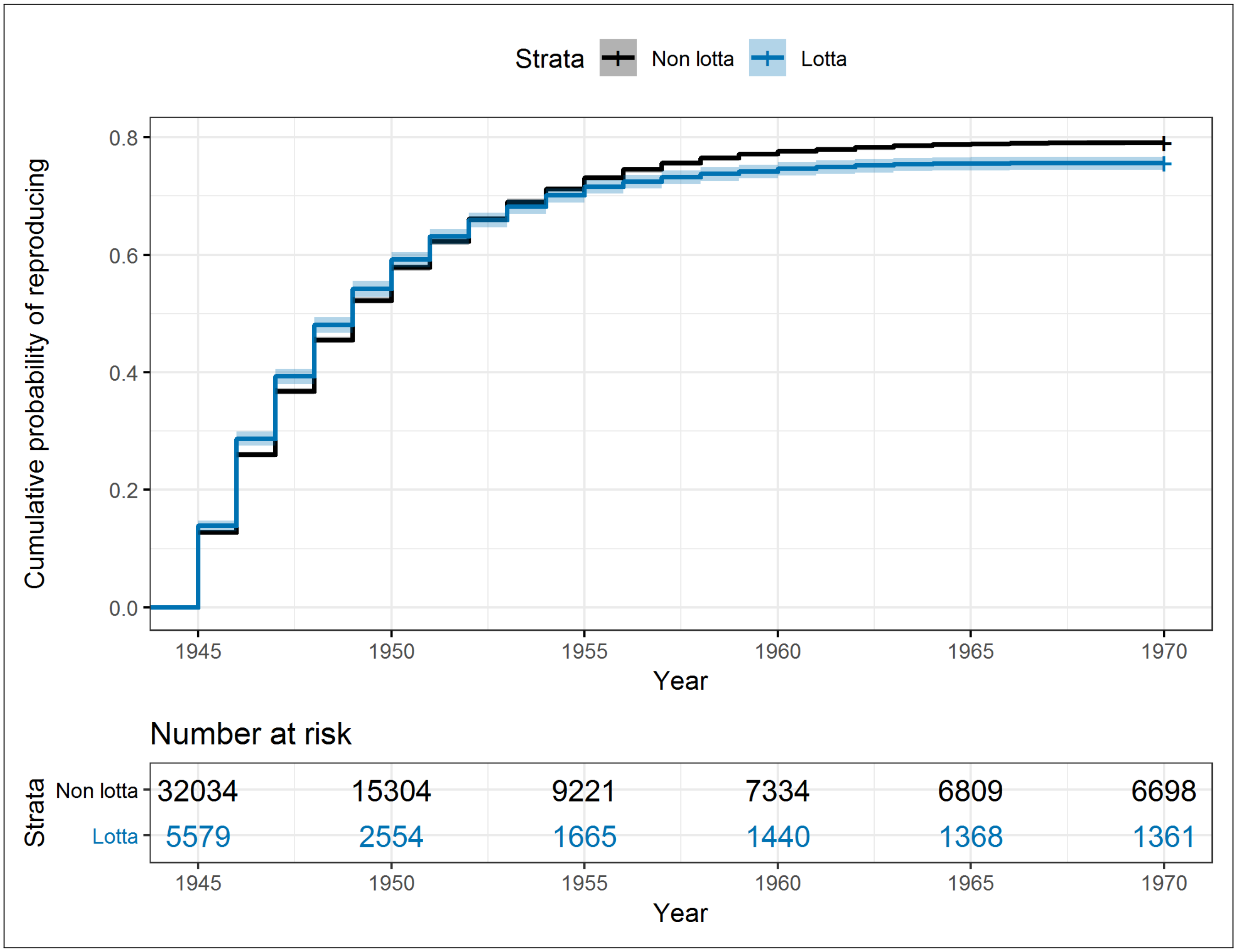

Supplementary Figure 5: Lottas are more likely to remain childless than non-Lottas. Cox survival model stratified by Lottas (blue) and Non Lottas (black). Top panel shows the cumulative probability of remaining childless for each successive year from 1945 (war ends) to 1970 (the year of the interview). Bottom panel shows samples group by 5 year periods.

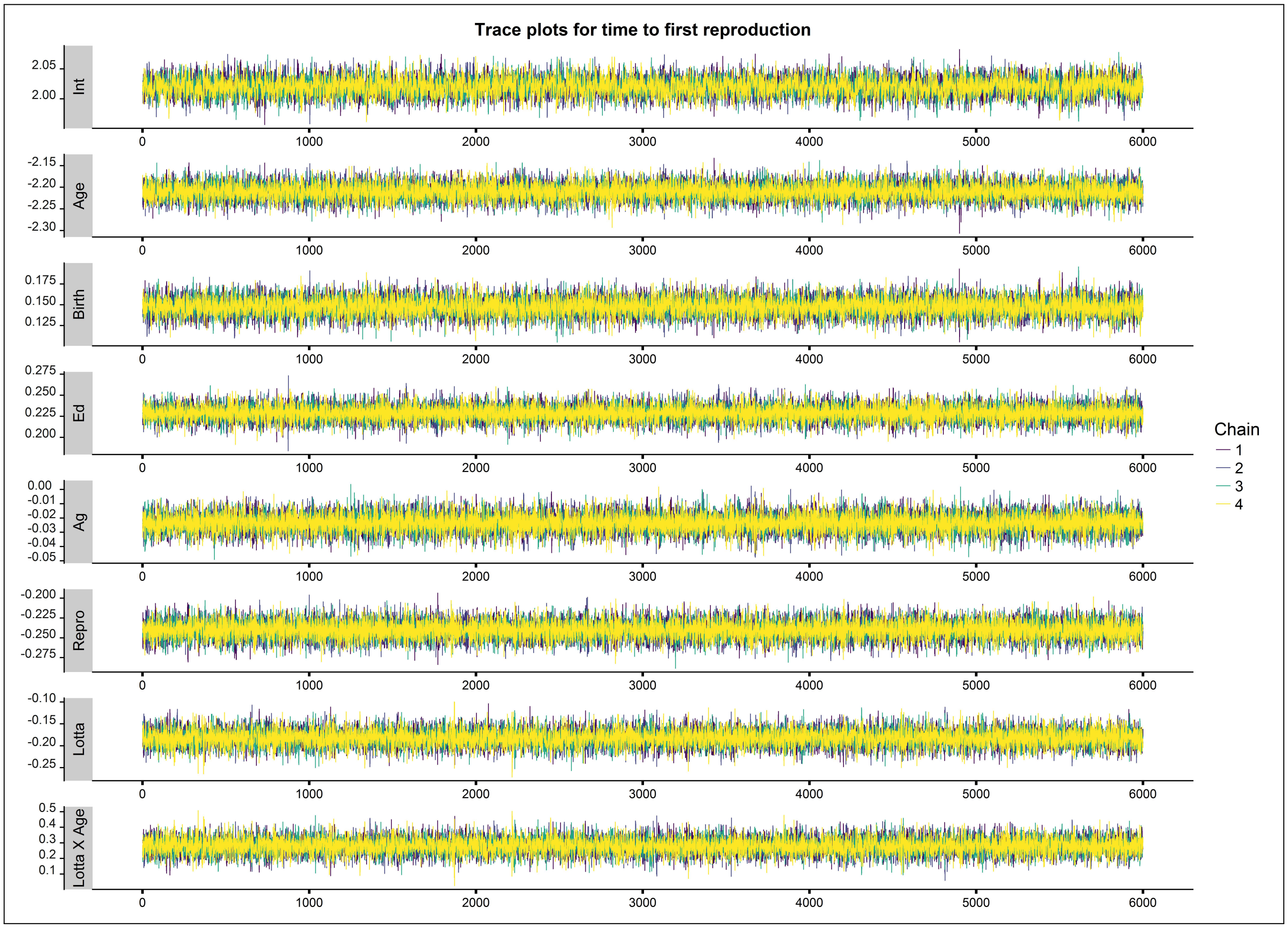

Supplementary Figure 6a: Trace plots for chains modeling time to first reproduction after the war using the full sample shows good mixing, convergence and stability across iterations (X-axis). This means that each chain converged on a similar posterior distribution which is generally seen as a characteristic of clean and healthy Markov chains (see Supplementary Figure 1a and Supplementary Table 1 (top right) for posterior distributions for all parameters entered into this model.)

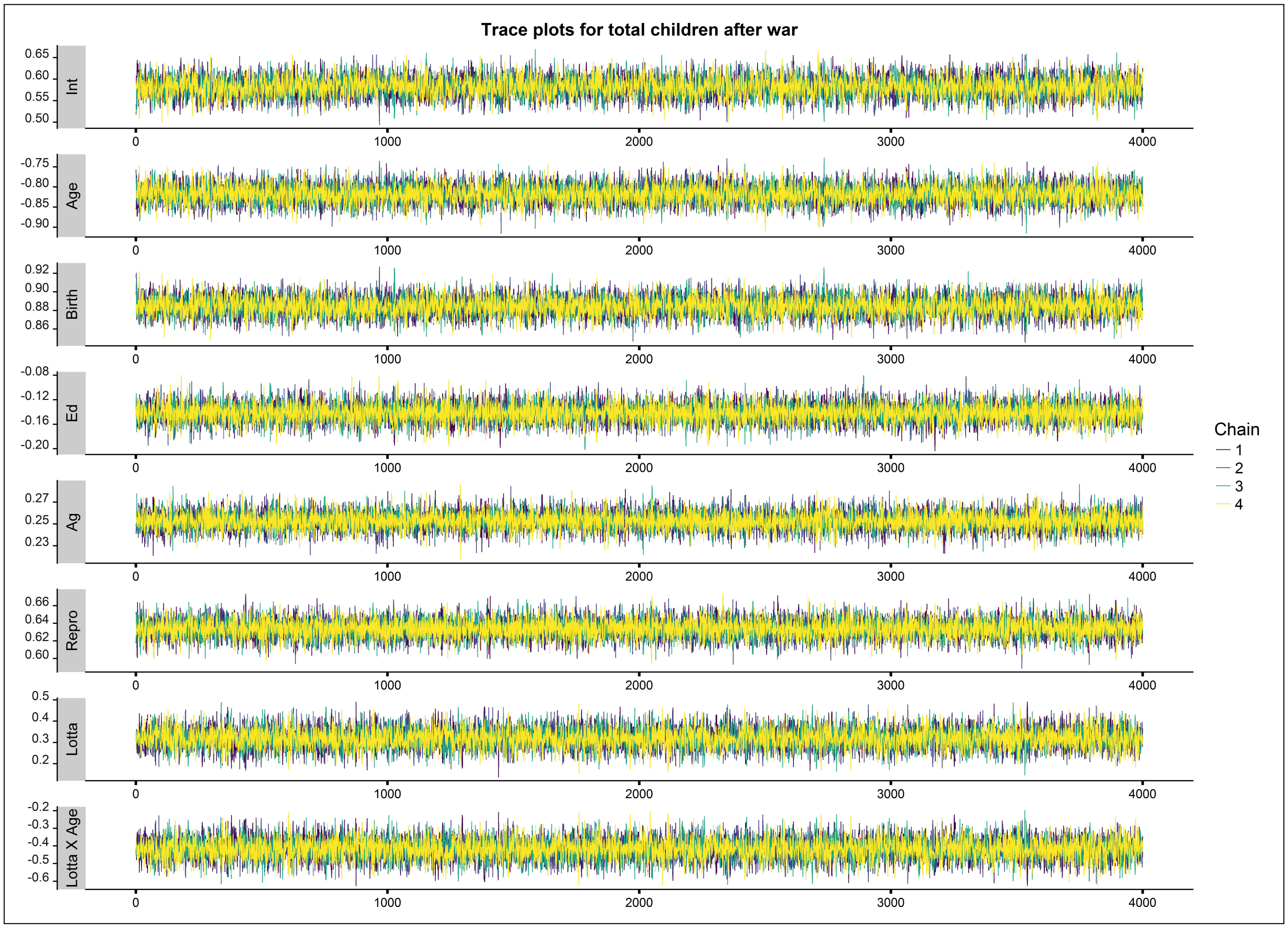

Supplementary Figure 6b: Trace plots for chains modeling total reproduction after the war using the full sample of shows good mixing, convergence and stability across iterations (X-axis). This means that each chain converged on a similar posterior distribution which is generally seen as a characteristic of clean and healthy Markov chains (see Supplementary Figure 1b and Supplementary Table 1 (bottom right) for posterior distributions for all parameters entered into this model.)

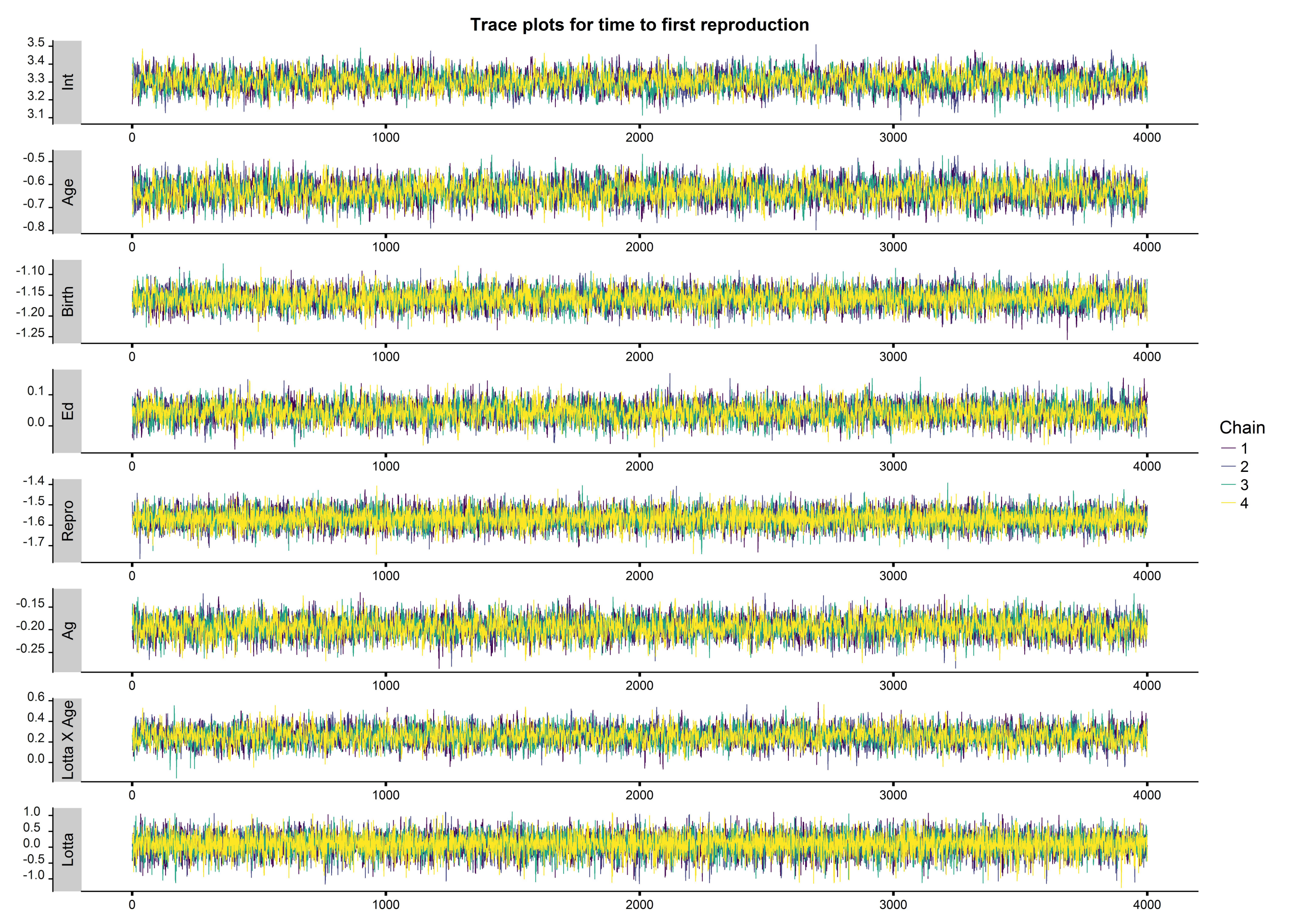

Supplementary Figure 7a: Trace plots for chains modeling time to first reproduction after the war using the sisters only sample shows good mixing, convergence and stability across iterations (X-axis). This means that each chain converged on a similar posterior distribution which is generally seen as a characteristic of clean and healthy Markov chains (see Supplementary Figure 2a and Supplementary Table 2 (top right) for posterior distributions for all parameters entered into this model.)

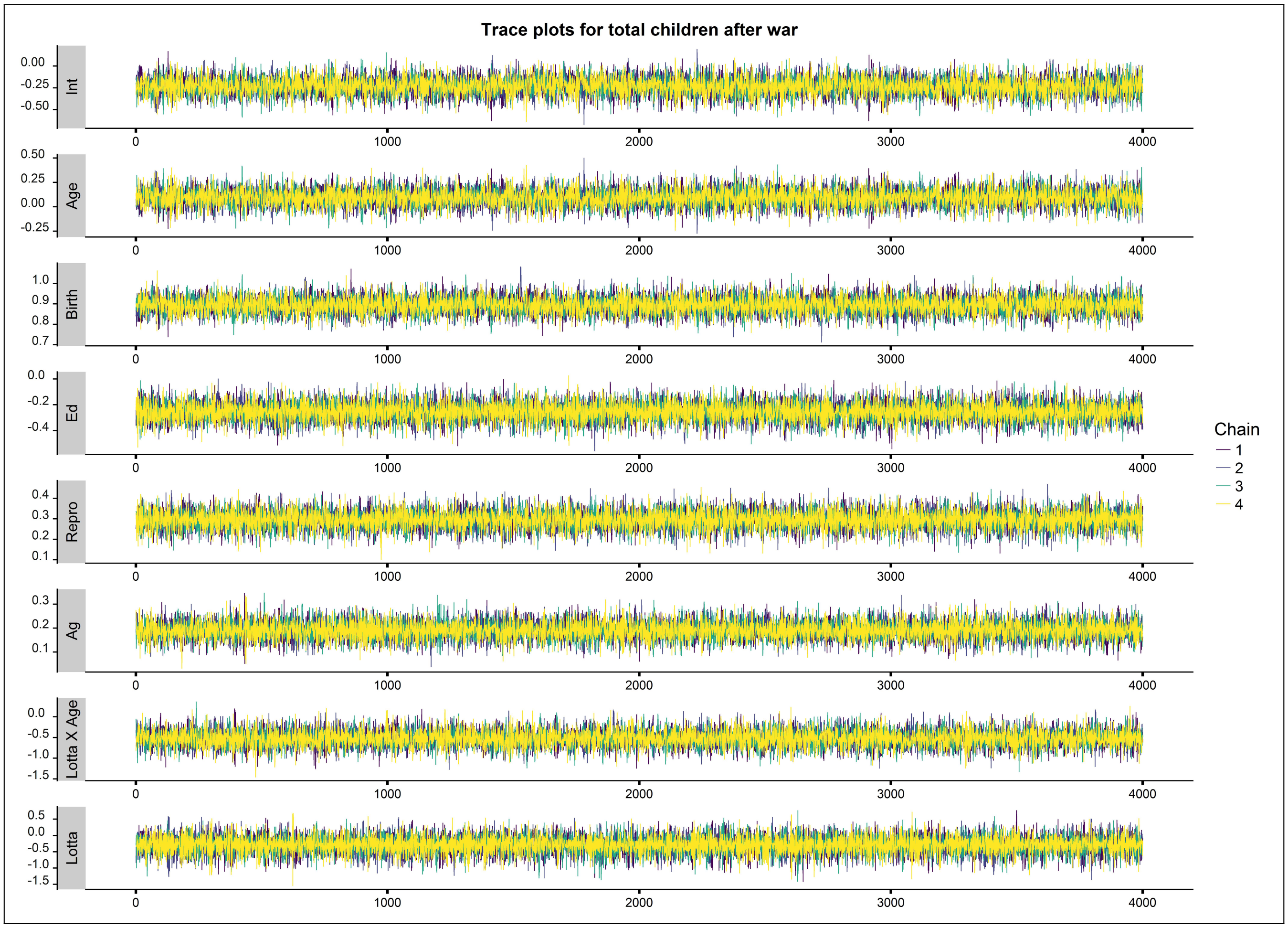

Supplementary Figure 7b: Trace plots for chains modeling total reproduction after the war using the sisters only sample shows good mixing, convergence and stability across iterations (X-axis). This means that each chain converged on a similar posterior distribution which is generally seen as a characteristic of clean and healthy Markov chains (see Supplementary Figure 2b and Supplementary Table 2 (bottom right) for posterior distributions for all parameters entered into this model.)

DV	Predictor	BEFORE THE WAR Mean	5% HDI	95% HDI	Odds Ratio	AFTER THE WAR Mean	5% HDI	95% HDI	Odds Ratio
**Time to first**	[Intercept]	2.71	2.69	2.74	15.0*	2.02	1.99	2.04	7.53*
**reproduction**	Age	0.42	0.39	0.45	1.52*	-2.20	-2.24	-2.18	0.11*
	Education	0.04	0.02	0.06	1.04*	0.23	0.21	0.24	1.25*
	Agriculture	-0.03	-0.04	-0.02	0.97*	-0.02	-0.03	-0.01	0.98*
[Before war	First child born after war	—-	—-	—-	—-	0.15	0.13	0.17	1.16*
N= 9,862	Reproduced within last 2 years	—-	—-	—-	—-	-0.24	-0.26	-0.22	-0.78*
After war	Lotta	-0.07	-0.14	0.00	0.93*	-0.18	-0.21	-0.15	0.83*
N= 31,613]	***Lotta X Age***	***0.07***	***0.01***	***0.13***	***1.10****	***0.28***	***0.20***	***0.37***	***1.32****
**Mean interbirth**	[Intercept]	0.58	0.56	0.61	1.79*	2.40	2.36	2.43	11.0*
**interval**	Age	-0.26	-0.28	-0.30	0.77*	-1.46	-1.50	-1.42	0.23*
	Education	0.02	0.01	0.04	1.02*	0.09	0.08	0.11	1.09*
	Agriculture	0.01	0.00	0.02	1.01	-0.07	-0.06	-0.08	0.93*
[Before war	First child born after war	—-	—-	—-	—-	0.08	0.06	0.09	1.08*
N=5,603	Reproduced within last 2 years	—-	—-	—-	—-	-0.07	-0.09	-0.06	0.93*
After war	Lotta	0.08	0.00	0.15	1.08	-0.09	-0.15	-0.02	0.91*
N= 31,613]	***Lotta X Age***	***-0.06***	***-0.12***	***0.00***	***0.94***	***0.06***	***0.01***	***0.11***	***1.06****
**Total**	[Intercept]	-1.23	-1.27	-1.19	0.29*	0.58	0.54	0.62	1.78*
**reproduction**	Age	1.74	1.67	1.82	5.7*	-0.82	-0.85	-0.78	0.44*
	Education	-0.11	-0.13	-0.09	0.89*	-0.14	-0.17	-0.12	0.87*
	Agriculture	0.19	0.18	0.20	1.21*	0.25	0.24	0.27	1.28*
Before war	First child born after war	-3.03	-3.18	-2.87	0.05*	0.88	0.97	0.90	2.41*
N= 37,613	Reproduced within last 2 years	—-	—-	—-	—-	0.63	0.72	0.65	1.88*
After war	Lotta	0.14	0.08	0.20	1.15*	0.32	0.24	0.40	1.38*
N= 37,613]	***Lotta X Age***	***-0.26***	***-0.33***	***-0.19***	***0.77****	***-0.42***	***-0.51***	***-0.32***	***0.66****

Supplementary Table 1: Parameter estimates, Highest Density Intervals (HDI's) and Odds ratios for factors affecting time to first reproduction (top panel), mean birth interval after the war (middle panel) and total reproduction (bottom panel) before (left side) and after (right side) the war for full sample. The parameter estimate of primary interest, the Lotta X Age interaction is both more pronounced and in the **hypothesized** direction after the war (i.e. positive or more positive for time to first reproduction and mean interbirth intervals and negative for total reproduction (right panels). *Parameter estimate 95% HDI does not overlap with zero.

		BEFORE THE WAR				AFTER THE WAR			
**DV**	**Predictor**	**Mean**	**5% HDI**	**95% HDI**	**Odds Ratio**	**Mean**	**5% HDI**	**95% HDI**	**Odds Ratio**
**Time to first**	[Intercept]	3.20	3.10	3.30	24.5*	3.44	3.29	3.59	31.2*
**reproduction**	Age	0.06	-0.02	0.14	1.06	-0.56	-0.74	-0.46	0.57*
	Education	-0.12	-0.18	-0.05	0.87*	0.12	0.06	0.19	1.27*
	Agriculture	-0.02	-0.04	0.00	0.98*	-0.25	-0.31	-0.18	0.78*
[Before war	First child born after war	—-	—-	—-	—-	-1.14	-1.22	-1.09	0.32*
N= 729	Reproduced within last 2 years	—-	—-	—-	—-	-1.33	-1.48	-1.19	0.26*
After war	Lotta	-0.06	-0.24	0.12	0.94	-0.36	-0.56	-0.16	0.70*
N= 2,272]	***Lotta X Age***	***0.06***	***-0.02***	***0.14***	***1.06***	***0.42***	***0.22***	***0.60***	***1.52****
**Mean interbirth**	[Intercept]	1.43	1.03	1.73	4.20*	1.22	1.16	1.28	3.39*
**interval**	Age	-0.81	-0.95	-0.67*	0.44	-0.32	-0.37	-0.27	0.73*
	Education	-0.06	-0.15	0.03	0.94*	0.11	0.03	0.19	1.12*
	Agriculture	0.00	-0.03	0.03	1.00	-0.05	-0.09	-0.01	0.95*
[Before war	First child born after war	—-	—-	—-	—-	0.11	0.03	0.19	1.12*
N=268	Reproduced within last 2 years	—-	—-	—-	—-	-0.02	-0.10	0.06	0.98
After war	Lotta	0.18	-0.42	0.78	1.20	-0.09	-0.15	-0.03	0.91*
N= 2,272]	***Lotta X Age***	***-0.09***	***-0.23***	***0.05***	***0.91***	***0.09***	***-0.01***	***0.19***	***1.09***
**Total**	[Intercept]	0.43	0.27	0.61	1.54*	-0.23	-0.40	-0.06	0.79*
**reproduction**	Age	0.12	-0.04	0.28	1.13	0.03	-0.05	0.23	1.03
	Education	0.03	-0.07	0.13	1.03	-0.25	-0.37	-0.13	0.78*
	Agriculture	-0.03	-0.09	0.03	0.97	0.19	0.13	0.25	1.21*
Before war	First child born after war	-0.71	-0.88	-0.54	0.49*	0.89	0.82	0.96	2.4*
N= 2,671	Reproduced within last 2 years	—-	—-	—-	—-	0.29	0.22	0.36	1.34*
After war	Lotta	-0.26	-0.64	0.12	0.77	0.32	0.00	0.67	1.38*
N= 2,671]	***Lotta X Age***	***0.31***	***-0.01***	***0.64***	***1.39***	***-0.52***	***-0.88***	***-0.20***	***0.59****

Supplementary Table 2: Parameter estimates, Highest Density Intervals (HDI's) and Odds ratios for factors affecting time to first reproduction (top panel), mean birth interval after the war (middle panel) and total reproduction (bottom panel) before (left side) and after (right side) the war for sisters only. The parameter estimate of primary interest, the Lotta X Age interaction is both more pronounced and in the **hypothesized** direction after the war (i.e. positive or more positive for time to first reproduction and mean interbirth intervals and negative for total reproduction (right panels).*Parameter estimate 95% HDI does not overlap with zero.

DV	Predictor	Hazard ratio	5% CI	95% CI	(Hazard ratio)
**Time to first**	Age	2.36	2.22	2.50	0.80
**reproduction**	Education	0.77	0.74	0.81	1.29
**after the war**	Agriculture	1.17	1.14	1.21	0.85
[N= 37,613]	First child born after war	4.73	4.58	4.89	0.21
	Reproduced within last 2 years	4.41	4.27	4.56	0.22
	Lotta	1.25	1.17	1.32	0.80
	Lotta X Age	0.51	0.44	0.59	1.95
**Mean birth**	Age	2.39	2.25	2.54	0.42
**interval**	Education	0.83	0.80	0.88	1.19
**after the war**	Agriculture	1.20	1.17	1.23	0.83
[N= 37,613]	First child born after war	4.72	4.56	4.87	0.21
	Reproduced within last 2 years	3.71	3.60	3.84	0.27
	Lotta	1.17	1.10	1.25	0.85
	Lotta X Age	0.55	0.47	0.63	1.82

Supplementary Table 3: Hazard ratios and 95% CI's from Cox survival analysis results for factors affecting time to first reproduction (top panel) and mean birth interval (bottom panel) using censored data (i.e. includes women who did not reproduce). Although inverse Hazard ratios (last column) are not directly analogous to the odd ratios generated by the Bayesian models shown in Supplementary Table 1, and therefore cannot be compared directly, the general conclusions from these models are the same.

DV	Predictor	Estimate	S.E.	p-value
**Never**	Intercept	-4.02	0.06	***
**Reproduced**	Education	0.48	0.04	***
[N=37,613]	Agriculture	-0.60	0.03	***
	Lotta	-1.00	0.17	***
	Age	0.10	0.001	***
	Lotta X Age	0.03	0.005	***

Supplementary Table 4: The impact of volunteering and age on the likelihood of never reproducing. Results from frequentist generalized linear model.

Lotta type	YOUNG - under age 25 More exposed to combat			YOUNG - under age 25 Less exposed to combat			Old - over age 24 More exposed to combat			Old - over age 24 Less exposed to combat		
	Mean	SE	N	Mean	SE	N	Mean	SE	N	Mean	SE	N
Time to reproduction	4.04	0.19	378	4.04	0.31	132	2.86	0.10	897	2.44	0.13	504
Interbirth intervals	4.12	0.20	378	4.36	0.53	378	3.27	0.17	897	2.95	0.21	504
Total reproduction	2.54	0.08	418	2.26	0.13	162	1.64	0.04	1273	1.42	0.05	727

Supplementary Table 5: Time to reproduction, interbirth intervals and overall reproduction after the war for different types of Lottas. Canteen workers, nurses and anti-aircraft volunteers were seen as more likely to be exposed to casualties and were categorized as 'More exposed to combat' while office workers and organizational volunteers were categorized as 'Less exposed to combat'.

Characteristics of volunteers and non-volunteers								
Trait	All Lottas		All Non-Lottas		Lotta sister		Non-Lotta sister	
*variables entered as covariates in models	Mean	SE	Mean	SE	Mean	SE	Mean	SE
Sample size	5,579	–	32,034	–	477	–	2,194	–
*Age in 1945	30.4	0.09	30.2	0.05	29.2	0.3	28.1	0.1
*Educated (%)	13	0.45	6.6	0.1	13	1.5	7	0.5
*Agricultural occupation (%)	20	0.4	28	0.2	18	2	29	1
*First child after war (%)	57.2	0.6	55.7	0.2	40	2	38	1
*Reproduced w/in 2 years (%)	13.8	0.4	13.4	0.2	10	1	12	0.5
**Birthplace population	13,758	197	13,336	69	11,831	719	12,187	356
Age at first birth before the war	25.1	0.1	24.3	0.05	26.5	0.5	26.2	0.2
Time to reproduction after the war	3.5	0.05	4.4	0.03	4.27	0.25	4.26	0.11
IBI before the war	2.86	0.06	3.03	0.02	2.59	0.31	3.12	0.15
IBI after the war	3.72	0.04	4.16	0.02	2.39	0.14	2.76	0.07
Total births before the war	1.75	0.03	2.31	0.02	1.66	0.08	1.56	0.04
Total births after the war	1.87	0.02	1.96	0.01	1.31	0.07	1.56	0.03
Number of siblings	4.51	0.06	4.8	0.03	5.03	0.2	4.81	0.2
Brothers	2.52	0.04	2.50	0.1	2.45	0.2	2.53	0.1
Sisters	1.26	0.01	1.25	0.005	2.20	0.2	2.20	0.2
Birth order	3.91	0.05	3.97	0.02	3.94	0.2	4.06	0.1
Returned to Karelia (%)	52	0.5	58	0.2	60	2	60	1

Supplementary Table 6: Comparison of Lottas and Non-Lottas.**Birthplace was entered as a random effect.

DV	Predictor	Mean	5% HDI	95% HDI	Odds ratio
**Time to first reproduction after the war** *[N= 19,091]*	[Intercept]	1.96	1.95	1.97	7.1
	Age	-2.00	-2.05	-1.95	0.13
	Education	0.24	0.21	0.26	1.27
	Agriculture	-0.03	-0.05	-0.02	0.97
	Reproduced within last 2 years	-0.35	-0.38	-0.32	0.70
	Lotta	-0.16	-0.20	-0.12	0.85
	Married before the war	0.03	0.00	0.06	1.03
	**Lotta X Married before the war**	**-0.13**	**-0.21**	**-0.04**	**0.88**
	**Lotta X Age**	**0.41**	**0.28**	**0.54**	**1.51**
**Mean interbirth interval after the war** *[N= 19,091]*	[Intercept]	1.77	1.79	1.78	5.9
	Age	-1.28	-1.33	-1.23	0.28
	Education	0.09	0.07	0.12	1.09
	Agriculture	-0.04	-0.06	-0.03	0.96
	Reproduced within last 2 years	-0.10	-0.13	-0.08	0.90
	Lotta	-0.06	-0.09	-0.02	0.94
	Married before the war	-0.04	-0.07	-0.01	0.96
	**Lotta X Married before the war**	**-0.08**	**-0.16**	**-0.01**	**0.92**
	**Lotta X Age**	**0.15**	**0.03**	**0.27**	**1.16**
**Total reproduction after the war** *[N= 22,113]*	[Intercept]	0.17	0.15	0.20	1.18
	Age	-0.74	-0.78	-0.70	0.48
	Education	-0.15	-0.18	-0.12	0.86
	Agriculture	0.26	0.24	0.28	11.28
	Reproduced within last 2 years	0.64	0.62	0.66	1.90
	Lotta	0.00	-0.06	0.07	1.00
	Married before the war	0.87	0.85	0.89	2.39
	**Lotta X Married before the war**	**0.09**	**0.04**	**0.15**	**1.09**
	**Lotta X Age**	**-0.29**	**-0.40**	**-0.17**	**0.75**

Supplementary Table 7: Parameter estimates, Highest Density Intervals (HDI's) and Odds ratios for factors affecting time to first reproduction (top panel), mean birth interval after the war (middle panel) and total reproduction (bottom panel) for individuals for whom wedding year was known. Here the parameter estimates of primary interest are the interactions between Lotta X Married before the war and Lotta X Age. Although the main effect of young Lottas having accelerated reproductive schedules remains (Lotta X Age 95% HDI does not overlap with zero) even after including whether or not women were married when the war began [dummy coded, married before 1940=1, married after 1939=0], it is important to note that the interaction between volunteering and when the women were married (Lotta X Married before the war) is also significant and runs in the opposite direction. This indicates that the effect of accelerated reproduction among young volunteers is more pronounced among women who were single when the war began.
