# Supplementary material for: Child volunteers in a women's paramilitary organization in World War II have accelerated reproductive schedules
Source: Nat Commun. 2020 May 12;11:2377. doi: 10.1038/s41467-020-15703-0 (PMC7217904; doi:10.1038/s41467-020-15703-0)
Supplement: Supplementary file 3 — Reporting Summary [file 41467_2020_15703_MOESM3_ESM.pdf]

nature research

Corresponding author(s): Robert Lynch
Last updated by author(s): Jan 15, 2020

Reporting Summary

Nature Research wishes to improve the reproducibility of the work that we publish. This form provides structure for consistency and transparency in reporting. For further information on Nature Research policies, see Authors & Referees and the Editorial Policy Checklist .

Statistics

For all statistical analyses, confirm that the following items are present in the figure legend, table legend, main text, or Methods section.

n/a	Confirmed	
[ ]	[x]	The exact sample size (*n*) for each experimental group/condition, given as a discrete number and unit of measurement
[ ]	[x]	A statement on whether measurements were taken from distinct samples or whether the same sample was measured repeatedly
[x]	[ ]	The statistical test(s) used AND whether they are one- or two-sided<br>*Only common tests should be described solely by name; describe more complex techniques in the Methods section.*
[ ]	[x]	A description of all covariates tested
[x]	[ ]	A description of any assumptions or corrections, such as tests of normality and adjustment for multiple comparisons
[ ]	[x]	A full description of the statistical parameters including central tendency (e.g. means) or other basic estimates (e.g. regression coefficient) AND variation (e.g. standard deviation) or associated estimates of uncertainty (e.g. confidence intervals)
[x]	[ ]	For null hypothesis testing, the test statistic (e.g. *F*, *t*, *r*) with confidence intervals, effect sizes, degrees of freedom and *P* value noted<br>*Give P values as exact values whenever suitable.*
[ ]	[x]	For Bayesian analysis, information on the choice of priors and Markov chain Monte Carlo settings
[ ]	[x]	For hierarchical and complex designs, identification of the appropriate level for tests and full reporting of outcomes
[x]	[ ]	Estimates of effect sizes (e.g. Cohen's *d*, Pearson's *r*), indicating how they were calculated

Our web collection on statistics for biologists contains articles on many of the points above.

Software and code

Policy information about availability of computer code

Data collection	Structured interviews of evacuees from Finnish Karelia during World War II were published in a four volume set called 'Siirtokarjalaisten tie'(Anon., 1970-1971). These records were compiled in an effort to record the lives of the Karelian evacuees during World War II. Over 300 individuals were trained to conduct these interviews, which took place between 1968 and 1970. During this time, an effort was made to locate everyone evacuated from Karelia during the war. Each entry in the published books lists the name, sex, date of birth, birthplace, occupation, year of marriage, reproductive records (name, sex, and date of birth of all children) and membership in various organizations, including Lotta Svärd. If they were married, the name, date of birth, birthplace and occupation of their spouse are also listed. These books were scanned with optical character recognition software, and additional software was developed (Kaira Core and Natural Language Processing software designed for use with the Finnish language) to digitize and extract these records (Loehr, J., Lynch, R., Mappes, J., Salmi, T., Pettay, J. and Lummaa, V., 2017) for a more details on data extraction methods and the construction of the database). Overall there are data on approximately 163,152 individuals, including spouses, but here we focus on a subset of 37,613 women for whom we had complete and credible records on their year of birth, place of birth, occupation, and years of birth of all their children. Of these individuals, 4,261 were listed as being both members of Lotta Svärd and who were between the ages of 12 and 40 in 1939. Finally, we were able to link some of the women in our data by their full names and exact dates of birth to a historical genealogy which uses digitized Finnish church records called 'Karjala-tietokanta' ("Katiha database," n.d.). We used these data to find a subset 3,190 full sisters who were all between the ages of 12 and 40 in 1940 (N=2,272 of whom reproduced). All R code for analysis, figures, and data selection is publicly available and can be found on Github (Lynch, 2019).<br><br>References<br><br>Loehr, J., Lynch, R., Mappes, J., Salmi, T., Pettay, J. and Lummaa, V. (2017). Newly Digitized MiKARELIA Database Reveals the Journeys of Second World War Forced Migrants from Finnish Karelia. Finnish Yearbook of Population Research.<br><br>Lynch. (2019). Lottas-LRS. Retrieved from https://github.com/robertlynch66/Lotta-LRS

Data analysis

To analyze the reproductive timing and lifetime reproductive success of Lotta Svärd volunteers, we used the rethinking package (R. McElreath, 2014) in R Studio 3.3.3 to run a generalized linear mixed-effects (GLMM) regression. Model fitting was performed using Hamiltonian Monte Carlo resampling, which draws samples from the posterior distribution, and was implemented with version 2.12 of Stan(Team, 2016). We used Bayesian inference for all statistical analyses, and assessed convergence of the Markov chains by inspection of the trace plots (see Supplementary Materials: Figures S4a-c), Gelman–Rubin Rˆ2, and an estimate of the effective number of samples. In a Bayesian framework, each model conditions data on prior probability distributions and uses Monte-Carlo methods to generate posterior distributions for each of the parameters. The priors are the initial probabilities for the values of each parameter. This type of analysis allows us to compare posterior distributions across occupational categories, age groups and educational backgrounds without relying on specific post-hoc tests(Richard McElreath, 2015) and averts the need to adjust for multiple comparisons(Gelman, Hill, & Yajima, 2012). We are also better able to visualize and interpret differences between parameter estimates relative to a specific value by reporting and displaying the entire posterior distribution for each predictor and showing the highest density intervals (HDI) to reveal the most credible values for each parameter estimate. Here, we assume that a parameter value was credibly different from the baseline if the 95% highest density intervals did not include zero.

To analyze how volunteering for Lotta Svärd impacted reproductive timing and reproductive success, we generated three models. Each was designed to predict three distinct outcomes: Model 1: Time to first birth after the war (N=31,613); Model 2: Mean birth intervals after the war (N=31,607); and Model 3: Total reproduction after the war (N=37,613) (see Supplementary Materials Table S1). In models 1 and 2, we initially included only women who had reproduced, as non-reproductive women cannot, by definition, have mean inter-birth intervals or time to first birth. Additional models were therefore developed to determine the models sensitivity to excluding non-reproductive women from models 1 and 2 (see Supplementary Materials: Table S2).

The predictor variables for all analyses were as follows: Age when the war ended in 1945, dummy variables encoding whether or not their occupation required an education (binary: 1=yes, 0=no), whether or not they were a farmer (binary: 1=farmer, 0=not a farmer), whether their first child was born after the war (binary: 1=yes, 0=no), whether or not they had given birth within the previous 2 years (binary: 1=yes, 0=no), whether or not they had volunteered for Lotta Svärd (binary: 1=yes, 0=no), and an interaction between their age in 1945 and whether or not they had volunteered. Finally, place of birth (N=991) was entered as a random effect into all models. Agriculture, and education were entered into the models because previous analyses have shown that these categories explain much of the variance in social status and social integration amongst this population(Lynch, R., Lummaa, V., Rotkirch, A., Danielsbacka, M., O'Brien, D. and Loehr, J., 2019). 'First child born after the war' was used to parse the effects of including women who had already had a child before 1945 and 'reproduced within the last two years' was entered to control for the reduced fertility of women following a birth (Howie & McNeilly, 1982). The interaction between volunteer status (Lotta) and a woman's age during the war was the predictor of interest.

Statistical analyses for all models were performed in R version 3.3.2 and Bayesian inference used to conduct analyses for Models 1-3 was carried out using the rstan package for R (Gelman, 2017 version 2.14.1) an interface to Stan which uses a Hamiltonian Monte Carlo sampler(Hoffman & Gelman, 2014). We used the rethinking R package (Richard McElreath, 2017 version 1.59)which includes convenience functions for building, sampling, and summarizing models with a Bayesian framework (Richard McElreath, 2015). The replicate models using all women, including non reproductives, used Cox proportional hazards regression models, implemented with the functions coxph and Surv from the survival package [version 2.44-1.1] (Therneau, 2015). This allows us to account for censored data - in this case, right censored at 25 years (the number of years from 1945 to the interviews). Though this may bias estimates upwards for older women, the level of censoring was similar between lottas and non-lottas. A generalized linear model was used to analyze the subset of individuals who we were able to link to the Katiha genealogical database ("Katiha database," n.d.). Only individuals with at least one sister were included in these models and mother id was entered as a random (clustering) variable. All the same fixed effects covariates used in the main models were also included.

References

McElreath, R. (2014). rethinking: Statistical Rethinking book package. R package version 1.391.

Team, S. D. (2016). Stan modeling language users guide and reference manual, version 2.14. 0. Technical Report: NAVTRADEVCEN. Naval Training Device Center.

McElreath, R. (2015). Statistical Rethinking. Texts in Statistical Science. CRC Press.

Gelman, A., Hill, J., & Yajima, M. (2012). Why We (Usually) Don't Have to Worry About Multiple Comparisons. Journal of Research on Educational Effectiveness, 5(2), 189–211.

Lynch, R., Lummaa, V., Rotkirch, A., Danielsbacka, M., O'Brien, D. and Loehr, J. (2019). Integration involves a trade-off between fertility and status for Finnish evacuees in World War II. Nature Human Behaviour,

Howie, P. W., & McNeilly, A. S. (1982). Effect of breast-feeding patterns on human birth intervals. Journal of Reproduction and Fertility, 65(2), 545–557.

Gelman, A. (2017. Rstan. Retrieved from https://mc-stan.org/users/interfaces/rstan.html

McElreath, R. (2017). Statistical rethinking. Retrieved from https://github.com/rmcelreath/rethinking

Therneau, T. (2015). A Package for Survival Analysis in S. version 2.38.

Katiha database. (n.d.). Retrieved May 2018, from http://www.karjalatk.fi/katiha/index.php

For manuscripts utilizing custom algorithms or software that are central to the research but not yet described in published literature, software must be made available to editors/reviewers. We strongly encourage code deposition in a community repository (e.g. GitHub). See the Nature Research guidelines for submitting code & software for further information.

Data

Policy information about availability of data

All manuscripts must include a data availability statement. This statement should provide the following information, where applicable:

- Accession codes, unique identifiers, or web links for publicly available datasets
- A list of figures that have associated raw data
- A description of any restrictions on data availability

All R code for analysis, figures, and data selection is publicly available and can be found on Github (Lynch, 2019).

Lynch. (2019). Lottas-LRS. Retrieved from https://github.com/robertlynch66/Lotta-LRS

Field-specific reporting

Please select the one below that is the best fit for your research. If you are not sure, read the appropriate sections before making your selection.

☐ Life sciences ☒ Behavioural & social sciences ☐ Ecological, evolutionary & environmental sciences

For a reference copy of the document with all sections, see nature.com/documents/nr-reporting-summary-flat.pdf

Behavioural & social sciences study design

All studies must disclose on these points even when the disclosure is negative.

Study description

This is an observational study which provides a quasi natural experiment on some of the factors which influence the reproductive scheduling of women who served in a paramilitary organization during World war II.

Research sample

Structured interviews of evacuees from Finnish Karelia during World War II were published in a four volume set called 'Siirtokarjalaisten tie'(Anon., 1970-1971). These records were compiled in an effort to record the lives of the Karelian evacuees during World War II. Over 300 individuals were trained to conduct these interviews, which took place between 1968 and 1970. During this time, an effort was made to locate everyone evacuated from Karelia during the war. Each entry in the published books lists the name, sex, date of birth, birthplace, occupation, year of marriage, reproductive records (name, sex, and date of birth of all children) and membership in various organizations, including Lotta Svärd. If they were married, the name, date of birth, birthplace and occupation of their spouse are also listed. These books were scanned with optical character recognition software, and additional software was developed (Kaira Core and Natural Language Processing software designed for use with the Finnish language) to digitize and extract these records (Loehr, J., Lynch, R., Mappes, J., Salmi, T., Pettay, J. and Lummaa, V., 2017) for a more details on data extraction methods and the construction of the database). Overall there are data on approximately 163,152 individuals, including spouses, but here we focus on a subset of 37,613 women for whom we had complete and credible records on their year of birth, place of birth, occupation, and years of birth of all their children. Of these individuals, 4,261 were listed as being both members of Lotta Svärd and who were between the ages of 12 and 40 in 1939. Finally, we were able to link some of the women in our data by their full names and exact dates of birth to a historical genealogy which uses digitized Finnish church records called 'Karjala-tietokanta' ("Katiha database," n.d.). We used these data to find a subset 3,190 full sisters who were all between the ages of 12 and 40 in 1940 (N=2,272 of whom reproduced). All R code for analysis, figures, and data selection is publicly available and can be found on Github (Lynch, 2019).

References

Loehr, J., Lynch, R., Mappes, J., Salmi, T., Pettay, J. and Lummaa, V. (2017). Newly Digitized MiKARELIA Database Reveals the Journeys of Second World War Forced Migrants from Finnish Karelia. Finnish Yearbook of Population Research.

Lynch. (2019). Lottas-LRS. Retrieved from https://github.com/robertlynch66/Lotta-LRS

Sampling strategy

This is a population based database and an attempt was made to interview all evacuees who were still alive in 1970. However, because some evacuees were no longer alive in 1970 some of the older evacuees are missing from our sample. Also the sample used was based on individuals for whom complete data was available (e.g. sex, age, occupation, birth place, and all their children).

Data collection

Each entry in the published books lists the name, sex, date of birth, birthplace, occupation, year of marriage, reproductive records (name, sex, and date of birth of all children), membership in various organizations including Lotta Svard and the years and names of all places where they have lived from birth until the time they were interviewed. If they were married, the name, date of birth, birthplace and occupation of their spouse are also listed. These books were scanned and software was developed (Kaira Core and Natural Language

Processing (NLP) software designed for use with the Finnish language) to digitize and extract the records (see Loehr et al (2017) for a detailed description of data extraction methods and the MiKARELIA database). We extracted the name, sex, year of birth, occupation, spouse, spouse's occupation, the names of all places they had lived and the years they moved, their year of marriage and their number of children.

References

Loehr, J., Lynch, R., Mappes, J., Salmi, T., Pettay, J. and Lummaa, V. (2017). Newly Digitized MiKARELIA Database Reveals the Journeys of Second World War Forced Migrants from Finnish Karelia. Finnish Yearbook of Population Research.

Timing	Interviews were conducted between 1968 and 1970
Data exclusions	Individuals with missing information on any of the variables of interest -- sex, age, occupation, spouse, birth place, and all their children-- were excluded from these analyses.
Non-participation	N/A
Randomization	Two groups and their combinations were delimited for these analyses in a quasi experimental design. These groups are women who 1) served in Lotta Svard and 2) women who did not serve in Lotta Svard.

Reporting for specific materials, systems and methods

We require information from authors about some types of materials, experimental systems and methods used in many studies. Here, indicate whether each material, system or method listed is relevant to your study. If you are not sure if a list item applies to your research, read the appropriate section before selecting a response.

Materials & experimental systems

n/a	Involved in the study
☒	☐ Antibodies
☒	☐ Eukaryotic cell lines
☒	☐ Palaeontology
☒	☐ Animals and other organisms
☒	☐ Human research participants
☒	☐ Clinical data

Methods

n/a	Involved in the study
☒	☐ ChIP-seq
☒	☐ Flow cytometry
☒	☐ MRI-based neuroimaging
